# The β_3_ Adrenergic Receptor Antagonist L-748,337 Attenuates Dobutamine-Induced Cardiac Inefficiency While Preserving Inotropy in Anesthetized Pigs

**DOI:** 10.1177/10742484211048762

**Published:** 2021-09-23

**Authors:** Lars Rødland, Leif Rønning, Anders Benjamin Kildal, Ole-Jakob How

**Affiliations:** 1 Cardiovascular Research Group, Institute of Medical Biology, Faculty of Health Sciences, 8016 UiT–The Arctic University of Norway, Tromsø, Norway; 2 Department of Anesthesiology and Intensive Care, 60519 University Hospital of North Norway, Tromsø, Norway

**Keywords:** inotrope, cardiac energetics, cardiac efficiency, myocardial oxygen consumption, β_3_ adrenergic receptor

## Abstract

Excessive myocardial oxygen consumption (MVO_2_) is considered a limitation for catecholamines, termed oxygen cost of contractility. We hypothesize that increased MVO_2_ induced by dobutamine is not directly related to contractility but linked to intermediary myocardial metabolism. Furthermore, we hypothesize that selective β_3_ adrenergic receptor (β_3_AR) antagonism using L-748,337 prevents this. In an open-chest pig model, using general anesthesia, we assessed cardiac energetics, hemodynamics and arterial metabolic substrate levels at baseline, ½ hour and 6 hours after onset of drug infusion. Cardiac efficiency was assessed by relating MVO_2_ to left ventricular work (PVA; pressure–volume area). Three groups received dobutamine (5 μg/kg/min), dobutamine + L-748,337 (bolus 50 μg/kg), or saline for time-matched controls. Cardiac efficiency was impaired over time with dobutamine infusion, displayed by persistently increased unloaded MVO_2_ from ½ hour and 47% increase in the slope of the PVA–MVO_2_ relation after 6 hours. Contractility increased immediately with dobutamine infusion (*dP*/*dt*_max_; 1636 ± 478 vs 2888 ± 818 mmHg/s, *P* < 0.05) and persisted throughout the protocol (2864 ± 1055 mmHg/s, *P* < 0.05). Arterial free fatty acid increased gradually (0.22 ± 0.13 vs 0.39 ± 0.30 mM, *P* < 0.05) with peak levels after 6 hours (1.1 ± 0.4 mM, *P* < 0.05). By combining dobutamine with L-748,337 the progressive impairment in cardiac efficiency was attenuated. Interestingly, this combined treatment effect occurred despite similar alterations in cardiac inotropy and substrate supply. We conclude that the extent of cardiac inefficiency following adrenergic stimulation is dependent on the duration of drug infusion, and β_3_AR blockade may attenuate this effect.

## Introduction

Dobutamine (Dob) is an inotrope used for treating patients with acute heart failure and cardiogenic shock.^1^ There has been conflicting evidence on the impact on myocardial oxygen consumption (MVO_2_) using this inotrope. The general understanding is that sympathomimetic drugs lead to myocardial oxygen wastage because of cAMP-induced increase in calcium handling.^2-4^ Contradictory results have shown that Dob does not induce oxygen wastage at clinically relevant doses.^5^ A limitation in that study is that the measurements was carried out shortly after the onset of drug infusion. This is relevant since Dob-induced myocardial oxygen wastage was seen in the subsequent dose escalating protocol.^5^ Thus, it is difficult to conclude whether the cardiac inefficiency is time dependent, dose dependent, or a combination. Several different protocols investigating Dob-induced changes in cardiac energetics have been done using various doses,^6-12^ but with the infusion lasting for a maximum of around 1 hour.

Dob stimulates primarily the β_1_ adrenergic receptor (AR) which in turn activates hormone-sensitive lipase leading to lipolysis in adipose tissue and higher circulating levels of free fatty acid (FFA). Altered substrate metabolism has been shown to influence MVO_2_ and cardiac efficiency. A negative impact on myocardial energetics following increased myocardial FFA utilization has been shown,^13,14^ also in the presence of isoproterenol.^15^ This is due to a higher oxygen use for basal metabolism with increased FFA utilization. The same effect has been described in pigs when infusing FFA intravenously, with a 48% increase in unloaded MVO_2_ compared to glucose infusion.^16^

A third AR, the β_3_AR, has been identified. The β_3_AR agonist BRL 37344 has a lipo-mobilizing effect from adipose tissue^17-19^ likely through elevation of intracellular cAMP levels.^20^ This is accompanied by a negative inotropic effect.^21,22^ Thus, the receptor can have a central role in modulating the metabolic effects of adrenergic stimulation. β_3_AR blocking with L-748,337 has been shown to give a positive inotropic effect,^23^ and in theory may inhibit lipolysis in adipose tissue during β_1_AR activation.

We hypothesize that Dob-induced myocardial oxygen wastage is time dependent and related to higher circulating levels of FFA over several hours, and may be counteracted by blockade of the β_3_AR using L-748,337.

## Methods and Materials

### Experimental Animals

Twenty-one castrated domestic pigs (*Sus scrofa domesticus*) weighing 30 ± 5 kg (mean ± standard deviation) were included. The animals were in quarantine for 5-7 days for environmental adaptation with free access to water and food, with only water available overnight before experiments. The use of experimental animals in this protocol was approved by the Norwegian Food Safety Authority, following Norwegian legislations for the use of animals in experimental research. The animal experiments were conducted in accordance with the Consensus Author Guidelines on Animal Ethics and Welfare for Veterinary Journals published by the International Association of Veterinary Editors, and all the investigations adhere to the ARRIVE Guidelines.

### Anesthesia and Surgical Instrumentation

Premedication using intramuscular injections of ketamine (20 mg/kg) (Pfizer AS, Norway), midazolam (1 mg/kg) (B.Braun, Germany) and atropine (1 mg) (Nycomed Pharma, Norway) was administered to all animals. Thereafter, anesthesia was induced through inhalation of 5% isoflurane gas (Abbot, USA) prior to endotracheal intubation. Intravenous injections of pentobarbital sodium (10 mg/kg) (Abbot, USA) and fentanyl (0.01 mg/kg) (Hameln Pharmaceuticals, Germany) were given through an ear vein cannula. Mechanical ventilation with an air-oxygen mixture (FiO_2_) of 60% was used and ventilation was monitored by capnography and arterial blood gases taken regularly. Through a central venous catheter placed in the left internal jugular vein, anesthesia was maintained throughout the experiments by continuous infusion of pentobarbital sodium (4.0 mg/kg/h), fentanyl (0.02 mg/kg/h) and midazolam (0.3 mg/kg/h). Heparin (2500 IU) (Leo, Denmark) and amiodarone (5 mg/kg) (Sanofi-Synthelabo, Sweden) were injected to prevent blood clotting on catheters and cardiac arrhythmia, respectively. The urinary bladder was drained by cystotomy. Introducer sheaths were placed in: 1) the right common carotid artery to allow placement of a 7 Fr multisegment Millar MPVS Ultra pressure–volume catheter (Millar, USA) in the left ventricle (LV), 2) the right femoral vein for placement of a 7 Fr balloon catheter (Sorin, Italy) to conduct preload reductions by inferior vena cava occlusions, and 3) the right internal jugular vein for placement of a Swan-Ganz catheter in the pulmonary artery via the right atrium and ventricle. A central arterial catheter (BD Secalon-T^TM^, Argon, Netherlands) was placed in the abdominal aorta through the left femoral artery for mean arterial pressure measurements. Median sternotomy was performed followed by removal of the pericardium and ligation of the hemiazygos vein. The left anterior descending coronary artery, the circumflex coronary artery, the right coronary artery and the pulmonary artery were dissected free from connective tissue to place transit-time ultrasonic flow probes (Medi-stim, Norway) to measure coronary blood flow (CBF) and cardiac output. Sonomicrometric crystals (Sonometrics Corp., Canada) for collection of dimension data were sutured in 3 regions of the myocardium: apically, and anteriorly and posteriorly in the base of the LV wall. A pediatric central venous catheter (Arrow 24G, eSutures, USA) placed in the great cardiac vein via the superior vena cava was used for blood sampling. The pressure–volume catheter was placed in the LV lumen via the carotid introducer, and its position verified by visualization of the pressure–volume loop. The 7 Fr balloon catheter was placed in the inferior vena cava via the femoral vein introducer, and vena cava occlusions were obtained to verify its position.

### Drug Dosage

Dobutamine (Sigma Aldrich) was administered as a continuous intravenous infusion (5 μg/kg/min). L-748,337 (Sigma Aldrich) was given as a bolus dose (50 μg/kg) based on previous studies to selectively block the β_3_AR.^23-25^

### Experimental Protocol

Following surgical preparation and positioning of instruments, all animals underwent approximately 45 minutes of hemodynamic stabilization. To maintain intravascular volume, sodium chloride solution 0.9% (20 ml/kg/h) (Fresenius Kabi) was infused throughout experiments.

Short axis end-systolic and end-diastolic endocardial diameter measurements were done by epicardial echocardiography (GE Vivid-I, KPI Healthcare, USA) for calibration of the sonomicrometric crystals dimension data. At baseline; venous, arterial and cardiac venous blood samples, and recordings of general hemodynamics were obtained. Thereafter, 6-8 recordings of cardiac function and MVO_2_ at different workloads were obtained by stepwise inflation of the vena cava balloon catheter. Finally, an abrupt vena cava occlusion was done for assessment of preload-recruitable stroke work. After baseline recordings, the animals were given either Dob (n = 9), Dob in combination with L-748,337 (Dob + L; n = 5), or vehicle substance (sodium chloride 0.9%; n = 7). The L-748,337 bolus in the Dob + L group was given simultaneously as Dob infusion was started. Stable hemodynamics, defined by stable heart rate and mean arterial pressure, was present at 30 minutes after start of drug infusions, and all measurements and recordings were repeated as described above. Six hours after initiation of drug infusion the final recordings were carried out. All animals were euthanized with an overdose of pentobarbital sodium.

### Calculations

LV end-systolic- and end-diastolic volume (ESV and EDV, respectively) at baseline, steady state hemodynamics, were calculated using the ellipsoid formula $V=\frac{\pi {\left(S_{endo}\right)}^{2}}{6*\;L_{endo}}$ ,^26^ where *V* is volume, and *S*_endo_ and *L*_endo_ are endocardial short axis diameter in end-systole and end-diastole, respectively, measured from epicardial echocardiography. To measure the ESV and EDV, the short- and long-axis sonomicrometric crystal signals were calibrated against ESV and EDV at baseline, and converted to a composite output using the Area Length (Bullet) formula,^27^ $\text{Volume}=\frac{5}{6}\text{*Area*Length}$ . Stroke work (SW) was calculated as $\text{SW}=\left(\left(\frac{P_{\text{max}\;}+\text{ESP}}{2}\right)\right)-\text{EDP})*\text{SV}$ , where *P*_max_ (mmHg) is the maximum pressure in the LV, ESP and EDP (mmHg) are end-systolic- and end-diastolic pressure in the LV, respectively, and SV is LV stroke volume. Pressure–volume area (PVA, J/beat/100 g) was calculated as $\text{PVA}=\frac{\text{SW}+\text{PE}}{\text{LVW}}\text{*}100$ , where LVW is LV weight and PE is potential energy calculated as $\text{PE}=\left(\frac{\text{ESP*}\left(\text{ESV}-V_{0}\right)}{2}-\frac{\text{EDP}*\left(\text{EDV}-V_{0}\right)}{4}\right)*0.000133$ , where *V*_0_ is the LV volume at zero pressure and 0.000133 (J/mmHg/ml) is a constant for energy production. MVO_2_ (J/beat/100 g) was calculated using the formula ${\text{MVO}}_{2}=\left(\frac{\frac{\text{LVCBF}*{\text{O}}_{2{\text{sat}}_{\text{Δ}}}*\text{Hb}*1.39}{\text{HR}}\;*\left(20.2\right)}{\text{LVW}}*100\right)$ , where LVCBF is LV CBF (ml/min), O_2_sat_▵_ is the arteriovenous difference in arterial and great cardiac vein oxygen saturations, Hb is hemoglobin (g/ml), 1.39 is a constant for ml O_2_/g Hb, HR is heart rate (beats/min), and 20.2 is a constant for energy production (J/ml O_2_).

### Data Recording and Analyses

LV function, hemodynamics, and dimension data from sonomicrometric crystals were recorded and analyzed using ADI LabChart (ADInstruments; Dunedin, New Zealand). Coronary artery and pulmonary trunk blood flow was recorded using transit-time flow probes (Medi-stim, Norway) with connecting software. LVCBF was estimated from global CBF (GCBF) and adjusted to LV weight (LVW) using the formula $\text{LVCBF}=\text{GCBF*}0.7\text{*}\left(\frac{100}{\text{LVW}}\right)$ .^28^

### Statistical Analyses

Calculations and statistical analyses were conducted using spreadsheet (Excel, Microsoft Office 2017), SPSS software statistics package (IBM, New York, USA) and GraphPad Prism (GraphPad, USA). Hemodynamics and metabolic data were analyzed within groups using a two-way mixed analysis of variance for repeated measures, and multiple comparisons were adjusted for by Bonferroni correction. The effects of drug interventions on the pool of linear regression lines of the PVA–MVO_2_ relation were evaluated using linear mixed model both for covariance analysis and to test if the slopes and y-intercepts differ between timepoints. The linear mixed model was conducted with MVO_2_ as the dependent variable, PVA as covariate, and animal identification, PVA, time and PVA*time as random and fixed effects. *P* < 0.05 was considered statistically significant.

## Results

### Hemodynamic Effects of Drug Infusion

**Figure 1.**
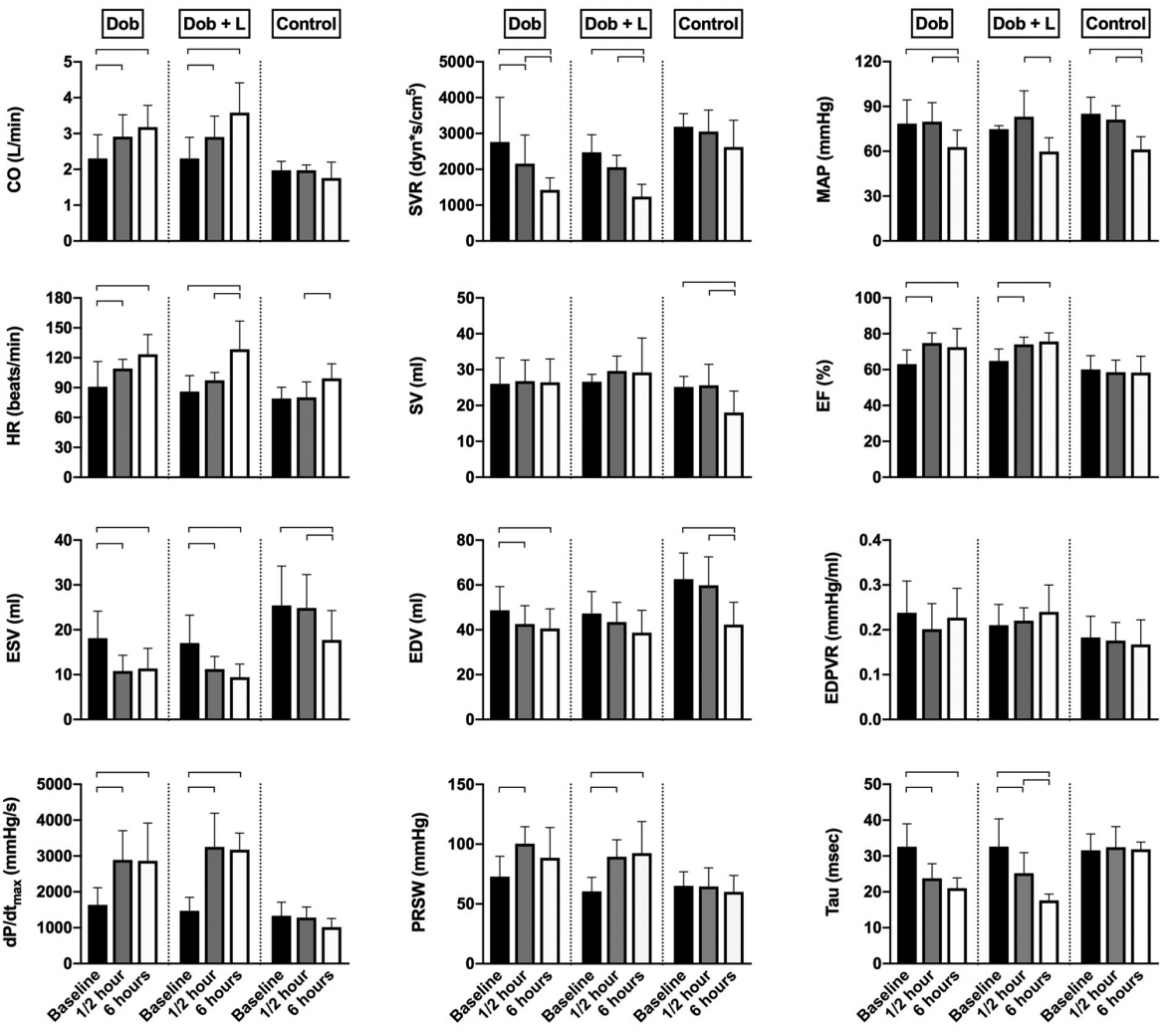
Hemodynamic data from healthy pigs. CO indicates cardiac output, measured using a flow probe on the pulmonary trunk; SVR, systemic vascular resistance, calculated from mean arterial pressure, central venous pressure in the vena cava and CO; MAP, mean arterial pressure measured using a vascular catheter in the abdominal aorta; HR, heart rate; SV, stroke volume calculated from CO and HR; EF, ejection fraction; End-systolic volume (ESV) and end-diastolic volume (EDV) calculated from sonomicrometric crystal signals calibrated by endocardial short axis diameter from epicardial echocardiography; EDPVR, end-diastolic pressure–volume relation given as the ratio of pressure divided by volume; *dP*/*dt*_max_, peak positive derivative of left ventricular pressure; PRSW, preload-recruitable stroke work, the slope of the relation between end-diastolic volume and stroke work; Tau, time constant of left ventricular isovolumetric relaxation calculated by Weiss method. Dob, dobutamine 5 μg/kg/min (n = 9); L, L-748,337 bolus 50 μg/kg (n = 5); Control, saline 0.9% (n = 7). Data were analyzed within groups using a two-way mixed ANOVA for repeated measures, and multiple comparisons were adjusted for by Bonferroni correction. Bars indicate mean values ± standard deviation. Brackets indicate statistically significant difference (*P* < 0.05).

Hemodynamic data are displayed in Figure 1. Baseline cardiac volumes varied between the groups, possibly because absolute volumetry is limited by the assumptions of accurate positioning of the sonomicrometric crystals and a perfect ellipsoid shape of the ventricle.

Dob gave an immediate increased cardiac output of 26% due to increased contractility, increased heart rate of 20% and a vasodilation with lowering of the systemic vascular resistance. Combining Dob with L-748,337 did not change cardiac inotropy and general hemodynamics. These immediate effects of the drugs were not diminished after 6 hours. Hemodynamics in time-matched controls showed preserved contractility and cardiac output. A progressive decline in mean arterial pressure throughout the protocol occurred in all 3 groups, which may have resulted from the cumulative effects of approximately 9 hours of deep general anesthesia in an open-chest experimental model.

### Cardiac Energetics and Metabolism

**Figure 2.**
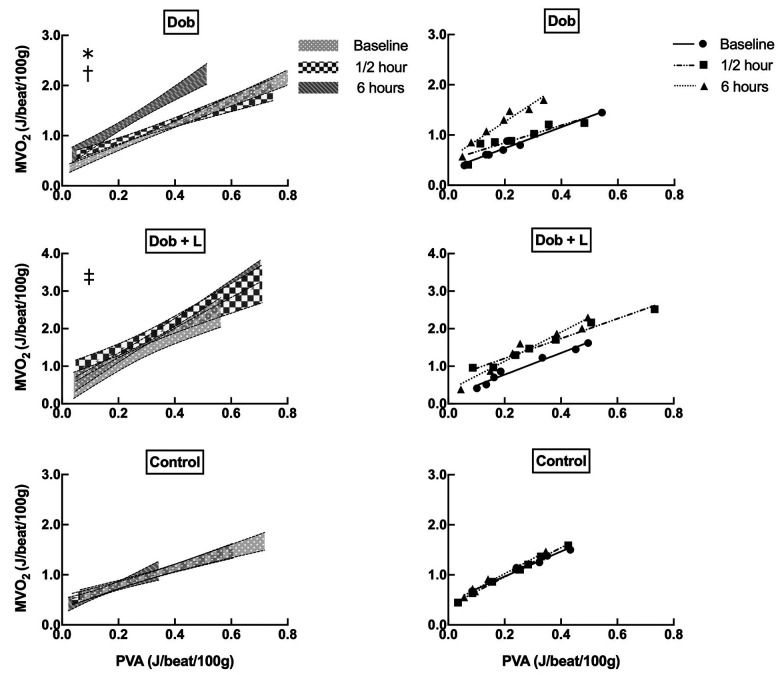
Left ventricular work–myocardial oxygen consumption relationship from healthy pigs. Left panels show 95% confidence intervals for pooled scatterplots from all experiments. Right panels show example data from one individual pig in each group. All data was obtained by preload reductions by vena cava occlusions using a balloon catheter. Dobutamine (Dob, 5 μg/kg/min, n = 9) impairs cardiac efficiency over time as displayed by an increase in y-intercept from baseline and increased slope after 6 hours (upper panel). Combining Dob with L-748,337 (L, bolus 50 μg/kg, n = 5) shows an increase in y-intercept after ½ hour compared to 6 hours, but after 6 hours y-intercept and slope is unchanged from baseline (middle panel). In time-matched controls (Control, saline 0.9%, n = 7) there is no change in cardiac efficiency for 6 hours (lower panel). MVO_2_, myocardial oxygen consumption; PVA, pressure–volume area. * *P* < 0.05, 6 hours vs baseline and ½ hour for slope; † *P* < 0.05, baseline vs ½ hour and 6 hours for y-intercept; ‡ *P* < 0.05, ½ hour vs 6 hours for y-intercept (linear mixed model analysis).

**Figure 3.**
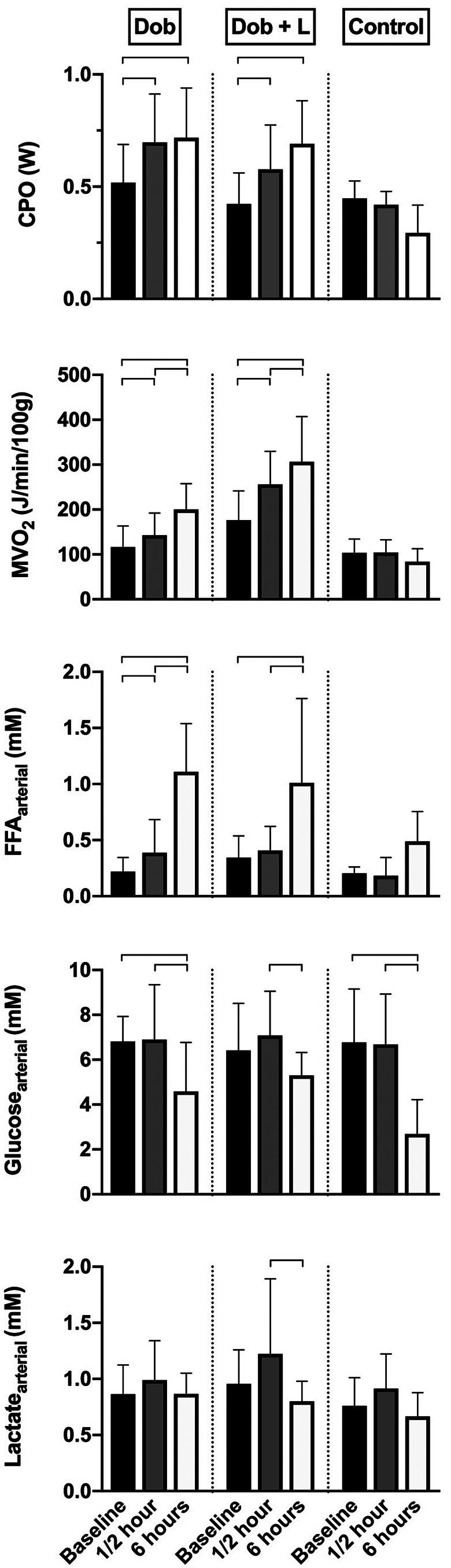
Metabolism data from healthy pigs. CPO, cardiac power output calculated as left ventricular developed pressure*cardiac output/451; MVO_2_, myocardial oxygen consumption; FFA, free fatty acid; Dob, dobutamine 5 μg/kg/min (n = 9); L, L-748,337 bolus 50 μg/kg (n = 5); Control, saline 0.9% (n = 7). Data were analyzed within groups using a two-way mixed ANOVA for repeated measures, and multiple comparisons were adjusted for by Bonferroni correction. Bars indicate mean values ± standard deviation. Brackets indicate statistically significant difference (*P* < 0.05).

Cardiac energetics are shown in Figure 2 and Table 1, and metabolic data in Figure 3. Cardiac efficiency was impaired over time with Dob infusion, displayed by persistently increased unloaded MVO_2_ from ½ hour and 47% increase in the slope of the PVA–MVO_2_ relation after 6 hours. When adding L-748,337 to Dob, y-intercept was higher after ½ hour compared to 6 hours, but at the end of the protocol neither y-intercept nor slope were significantly changed from baseline. Arterial FFA concentration was immediately elevated and consistently rose throughout 6 hours of Dob infusion, independently of the presence of the β_3_AR blocker. Both with Dob alone and in combination with L-748,337, steady state MVO_2_ increased over time of drug infusion. In time-matched controls, steady state LV work and MVO_2_ were matched during the entire protocol, and arterial FFA levels increased. When analyzing scatterplots for PVA–MVO_2_ there was no observed oxygen wastage in controls.

**Table 1.** Left Ventricular Energetics.^a^

	Baseline	½ hour	6 hours
Dobutamine 5 μg/kg/min, n = 9			
Slope	2.31 ± 0.13^b^	1.89 ± 0.20^b^	3.40 ± 0.33^c^
y-intercept	0.30 ± 0.04^b^	0.52 ± 0.06^c^	0.49 ± 0.09^c^
* R* ^2^	0.94 ± 0.03	0.90 ± 0.08	0.95 ± 0.04
Dobutamine 5 μg/kg/min			
+ L-748,337 50 μg/kg bolus, n = 5			
Slope	3.49 ± 0.58	3.39 ± 0.50	4.51 ± 0.31
y-intercept	0.35 ± 0.19	0.71 ± 0.16^b^	0.29 ± 0.10
* R* ^2^	0.94 ± 0.05	0.97 ± 0.01	0.93 ± 0.04
Control, saline 0.9%, n = 7			
Slope	1.71 ± 0.20	1.76 ± 0.20	2.18 ± 0.45
y-intercept	0.47 ± 0.07	0.45 ± 0.06	0.36 ± 0.08
* R* ^2^	0.95 ± 0.04	0.96 ± 0.05	0.93 ± 0.06

^a^ Slope and y-intercept of the left ventricular pressure–volume area (PVA)–myocardial oxygen consumption (MVO_2_) relationship over a broad range of workloads in healthy pigs. 1/slope is a measure of contractile efficiency (work-dependent oxygen consumption), whereas the y-intercept indicates MVO_2_ (J/beat/100 g) in the unloaded ventricle used for excitation-contraction coupling and basal metabolism. *R* ^2^ is the regression coefficient from the individual pigs. The data is presented as mean ± standard deviation.

^b^ *P* < 0.05, vs 6 hours (linear mixed model analysis).

^c^ *P* < 0.05, vs baseline (linear mixed model analysis).

## Discussion

### Main Finding

In this study, 6 hours of Dob infusion impaired cardiac efficiency as measured by a leftward shift of the PVA–MVO_2_ relationship (Figure 2). As cardiac inefficiency progressed throughout the protocol, this suggests a time dependent myocardial oxygen wastage by sympathomimetic drugs that is not related to the inotropic state of the heart. Furthermore, the results suggest that specific blockade of the β_3_AR attenuates this inefficiency without affecting the inotropic effect of Dob.

The PVA–MVO_2_ framework to assess cardiac energetics was introduced by Suga.^2,29^ This framework allows to differentiate work-dependent and work-independent energy consumption in the LV. The cost of basal metabolism and excitation-contraction coupling make up the work-independent MVO_2_, given as the y-intercept. The present study confirms the Suga concept of oxygen cost of contractility^30^ as seen by a concomitant elevation of inotropy and the y-intercept after ½ hour in both groups receiving Dob (Figure 1 and Table 1, respectively). This finding suggests that specific β_3_AR blockade does not interact with the inotrope signaling pathway and, thus, does not alter the oxygen cost of contractility in the healthy pig. However, the slope which represents work-dependent MVO_2_ was only increased after 6 hours in the Dob group. Thus, only Dob as monotherapy showed a clinically relevant impairment in cardiac efficiency at prolonged drug infusion. This decrement in efficiency is evident in Figure 2 as a substantial leftward shift in the PVA–MVO_2_ relationship after 6 hours. Measuring cardiac energetics using the PVA–MVO_2_ over a broad range of workloads is of great importance. This because the relationship of cardiac work (e.g. PVA) and energy consumption (MVO_2_) always has a positive y-intercept. Thus, a crude ratio of these 2 indexes as a measure of efficiency will erroneously favor the heart with the highest workload.^31^

Activation of the β_1_AR increases arterial levels of FFA, and the subsequent disproportional increased MVO_2_ due to increased FFA utilization for energy production has been described earlier.^13^ A theoretical total metabolic shift from 100% glucose oxidation to 100% FFA oxidation will increase unloaded MVO_2_ by 11%.^32^ But Mjøs O.D^13^ showed 26% increase in MVO_2_ when infusing intralipid in intact dogs, suggesting additional mechanisms for the high oxygen consumption. An unbalanced energy production with high intracellular FFA levels with an increased palmitate oxidation to oxygen consumption ratio^33^ and a futile triacylglycerol-fatty acid cycle^34^ have been proposed. Also, Borst et al^35^ reported fatty acid-induced uncoupling of oxidative phosphorylation without increased ATP production despite higher oxygen consumption. There is a marked energetical advantage with glucose as substrate for energy production,^14,16,36^ with several studies supporting the detrimental effects on myocardial energetics following increased use of FFA as substrate.^15,37^ Thus, therapies that lower the plasma FFA levels seems expedient, especially in the ischemic heart where CBF is considered a limiting factor. It is important to notice that serum glucose levels progressively declines throughout the protocol in all groups (Figure 3), which may have contributed to the decrease in cardiac efficiency by favoring myocardial FFA utilization via the Randle cycle.^38^

Myocardial oxygen wastage induced by inotropes has several potential causes. Increased calcium transients in myocardial cells has been shown to cause wastage due to higher energy expenditure for excitation-contraction coupling such as that reported using catecholamines as well as another inotrope (OPC 8212; phosphodiesterase 3 inhibitor).^30^ The changes in intracellular calcium occur immediately after β_1_AR stimulation as reflected by the *dP*/*dt*_max_ value in the present study. This may explain the surplus MVO_2_ seen after ½ hour of Dob infusion, but not the progressive disproportionate increase in work-dependent MVO_2_ as seen in the following 6 hours.

The progressive myocardial oxygen wastage is not linked to contractile state, but our data do not show whether it is inotropy, increased plasma concentrations of FFA, both those variables, or other factors that is causing the impaired cardiac efficiency following Dob infusion. Korvald et al^16^ showed acute myocardial oxygen wastage with a parallel upward shift of the PVA–MVO_2_ relation after high dose FFA giving around 3-fold the plasma concentrations induced by Dob in the present study. This was compared with glucose-insulin-potassium infusion. Further studies are warranted to investigate the effect of a moderate to high increase in plasma FFA on cardiac efficiency for 6 hours or more.

### β_3_ Adrenergic Receptor Antagonism

The L-748,337 dose was chosen to selectively block the β_3_AR, and a clear inotropic effect is not seen in this present study. Although the percentage increase in *dP*/*dt*_max_ during the first ½ hour of dobutamine infusion was larger when combined with L-748,337 treatment, the study was not designed to test the effect of L-748,337 alone. In heart failure the β_1_AR is desensitized by chronic adrenergic stimulation, while the lack of β_3_AR desensitization may contribute to worsening of cardiac function.^39^ Thus, a positive inotropic effect of L-748,337 is not expected in this model using healthy juvenile pigs when dobutamine is present. Systemic vascular resistance similarly was unaffected by L-748,337, which is surprising considering the compound is thought to inhibit the NO system.^40^ In particular since a previous study using the same strain of animals has shown high sensitivity to NOS blockade by L-NAME.^41^

The changes in blood levels of FFA, glucose and lactate (Figure 3) are similar within the 2 groups with Dob infusion. This may suggest a minimal effect on blocking lipolysis at the chosen doses of L-748,337. Considering that the myocardial oxygen wasting effect after 6 hours was observed only when Dob was the sole drug, and that the metabolic substrate supply was similar in both those groups, the question of a direct cardiac effect of L-748,337 arises. We were not able to detect differences in myocardial uptake of the metabolic substrates due to large variations in the samples. Thus, changes in myocardial substrate utilization using these drugs should be further explored. The β_3_AR has different characteristics than the β_1_AR and the β_2_AR, i.e. on G-proteins.^42^ Also, it is upregulated in failing myocardium^21^ and in cells exposed to chronic adrenergic stimulation,^43^ which is opposite to the β_1_AR and β_2_AR lower density in failing human hearts,^44^ suggesting that a protective role is feasible.

### Further Implications

The open-chest model is gold standard methodology to investigate cardiac energetics. In general, the clinical perspective is more applicable to a closed-chest situation, and further studies of this combination treatment for acute heart failure are warranted. Our main finding with substantial myocardial oxygen wastage after 6 hours of Dob infusion is of importance due to the use of this inotrope in patients to treat acute heart failure^1^ and septic shock.^45^ Recommended doses to treat acute heart failure range from 1 to 20 μg/kg/min^46^ with the infusion often lasting for several days.^47,48^ Myocardial oxygen wastage is considered an adverse effect when administering Dob in patients, and β_1_AR stimulation in adipose tissue increases arterial FFA levels which is associated with worsened mechanical function in ischemic hearts.^49^ In one study of patients with dilated cardiomyopathy, carvedilol, a non-selective βAR antagonist and α_1_AR antagonist, increased ejection fraction from 26% to 37% after 3 months of treatment.^50^ This improvement of LV pump function was accompanied by a 57% reduction in myocardial use of FFA in these patients. Other clinical studies have shown beneficial effects of treatment with glucose-insulin-potassium infusion in post-operative cardiac failure.^51,52^

Based on the evidence of the detrimental effect of relatively high FFA utilization in the heart under certain conditions, the β_3_AR antagonists can potentially improve cardiac function and increase contractile indexes without increased lipolysis when treating ischemic acute heart failure. In the present study, β_3_AR blocking was unable to restrict the Dob-mediated increase in plasma FFA and did neither change hemodynamics or contractility in healthy pigs. Thus, effects of β_3_AR blocking on hemodynamics and cardiac energetics in ischemic acute heart failure needs to be investigated.

## Conclusions

These data suggest that the extent of dobutamine-induced myocardial oxygen wastage is time dependent for a minimum of 6 hours. This finding supports earlier studies showing that myocardial oxygen wastage seen as an adverse effect of sympathomimetic drugs can be related to a higher exposure of FFA to the cardiomyocytes, but our data does not show any causality regarding this. The β_3_AR antagonist L-748,337 attenuates the progressive dobutamine-induced cardiac inefficiency, without affecting inotropy, general hemodynamics or substrate supply.

## References

[1] Ponikowski P Voors AA Anker SD , et al. 2016 ESC guidelines for the diagnosis and treatment of acute and chronic heart failure: The task force for the diagnosis and treatment of acute and chronic heart failure of the European Society of Cardiology (ESC). Developed with the special contribution of the Heart Failure Association (HFA) of the ESC. Eur J Heart Fail. 2016;18(8):891–975. 27207191 10.1002/ejhf.592

[2] Suga H . Ventricular energetics. Physiolo Rev. 1990;70(2):247–277. 10.1152/physrev.1990.70.2.247 2181496

[bibr3-10742484211048762] Sarnoff SJ Braunwald E Welch GH Jr Case RB Stainsby WN Macruz R . Hemodynamic determinants of oxygen consumption of the heart with special reference to the tension-time index. Am J Physiol. 1958;192(1):148–156. 13498167 10.1152/ajplegacy.1957.192.1.148

[4] Ruf T Hebisch S Gross R Alpert N Just H Holubarsch C . Modulation of myocardial economy and efficiency in mammalian failing and non-failing myocardium by calcium channel activation and beta-adrenergic stimulation. Cardiovasc Res. 1996;32(6):1047–1055. 9015407 10.1016/s0008-6363(96)00157-5

[5] Muller S How OJ Jakobsen O , et al. Oxygen-wasting effect of inotropy: is there a need for a new evaluation? An experimental large-animal study using dobutamine and levosimendan. Circ Heart Fail. 2010;3(2):277–285. 20018954 10.1161/CIRCHEARTFAILURE.109.865519

[6] Sundram P Reddy HK McElroy PA Janicki JS Weber KT . Myocardial energetics and efficiency in patients with idiopathic cardiomyopathy: response to dobutamine and amrinone. Am Heart J. 1990;119(4):891–898. 2321508 10.1016/s0002-8703(05)80328-x

[bibr7-10742484211048762] Hata K Goto Y Futaki S , et al. Mechanoenergetic effects of pimobendan in canine left ventricles. Comparison with dobutamine. Circulation. 1992;86(4):1291–1301. 1394935 10.1161/01.cir.86.4.1291

[bibr8-10742484211048762] Grandis DJ MacGowan GA Koretsky AP . Comparison of the effects of ORG 30029, dobutamine and high perfusate calcium on function and metabolism in rat heart. J Mol Cell Cardiol. 1998;30(12):2605–2612. 9990532 10.1006/jmcc.1998.0817

[bibr9-10742484211048762] Yi KD Downey HF Bian X Fu M Mallet RT . Dobutamine enhances both contractile function and energy reserves in hypoperfused canine right ventricle. Am J Physiol Heart Circ Physiol. 2000;279(6):H2975–H2985. 11087255 10.1152/ajpheart.2000.279.6.H2975

[bibr10-10742484211048762] Saupe KW Eberli FR Ingwall JS Apstein CS . Metabolic support as an adjunct to inotropic support in the hypoperfused heart. J Mol Cell Cardiol. 2001;33(2):261–269. 11162131 10.1006/jmcc.2000.1297

[bibr11-10742484211048762] Nakajima-Takenaka C Sakata S Kato S , et al. Detrimental effects after dobutamine infusion on rat left ventricular function: mechanical work and energetics. Exp Physiol. 2005;90(4):635–644. 15849228 10.1113/expphysiol.2005.030460

[12] Rider OJ Francis JM Ali MK , et al. Effects of catecholamine stress on diastolic function and myocardial energetics in obesity. Circulation. 2012;125(12):1511–1519. 22368152 10.1161/CIRCULATIONAHA.111.069518

[13] Mjos OD . Effect of free fatty acids on myocardial function and oxygen consumption in intact dogs. J Clin Investig. 1971;50(7):1386–1389. 5090055 10.1172/JCI106621 PMC292076

[14] How OJ Aasum E Kunnathu S Severson DL Myhre ES Larsen TS . Influence of substrate supply on cardiac efficiency, as measured by pressure-volume analysis in ex vivo mouse hearts. Am J Physiol Heart Circ Physiol. 2005;288(6):H2979–H2985. 15764683 10.1152/ajpheart.00084.2005

[15] Mjos OD . Effect of inhibition of lipolysis on myocardial oxygen consumption in the presence of isoproterenol. J Clin Investig. 1971;50(9):1869–1873. 5564394 10.1172/JCI106679 PMC292113

[16] Korvald C Elvenes OP Myrmel T . Myocardial substrate metabolism influences left ventricular energetics in vivo. Am J Physiol Heart Circ Physiol. 2000;278(4):H1345–H1351. 10749732 10.1152/ajpheart.2000.278.4.H1345

[17] Reverte M Rivas-Cabanero L . Effects of the beta 3-adrenoceptor agonist BRL 37344 on lipomobilization and plasma glucose levels in conscious fasted rabbits. Can J Physiol Pharmacol. 1996;74(3):251–256. 8773403

[bibr18-10742484211048762] Sasaki N Uchida E Niiyama M Yoshida T Saito M . Anti-obesity effects of selective agonists to the beta 3-adrenergic receptor in dogs. I. The presence of canine beta 3-adrenergic receptor and in vivo lipomobilization by its agonists. J Vet Med Sci. 1998;60(4):459–463. 9592718 10.1292/jvms.60.459

[19] Galitzky J Reverte M Carpene C Lafontan M Berlan M . Beta 3-adrenoceptors in dog adipose tissue: studies on their involvement in the lipomobilizing effect of catecholamines. J Pharmacol Exp Ther. 1993;266(1):358–366. 8101220

[20] Fisher MH Amend AM Bach TJ , et al. A selective human beta3 adrenergic receptor agonist increases metabolic rate in rhesus monkeys. J Clin Investig. 1998;101(11):2387–2393. 9616210 10.1172/JCI2496 PMC508828

[21] Napp A Brixius K Pott C , et al. Effects of the beta3-adrenergic agonist BRL 37344 on endothelial nitric oxide synthase phosphorylation and force of contraction in human failing myocardium. J Card Fail. 2009;15(1):57–67. 19181295 10.1016/j.cardfail.2008.08.006

[22] Gauthier C Tavernier G Charpentier F Langin D Le Marec H . Functional beta3-adrenoceptor in the human heart. J Clin Investig. 1996;98(2):556–562. 8755668 10.1172/JCI118823 PMC507461

[23] Morimoto A Hasegawa H Cheng HJ Little WC Cheng CP . Endogenous beta3-adrenoreceptor activation contributes to left ventricular and cardiomyocyte dysfunction in heart failure. Am J Physiol Heart Circ Physiol. 2004;286(6):H2425–H2433. 14962832 10.1152/ajpheart.01045.2003

[bibr24-10742484211048762] Moniotte S Balligand JL . Potential use of beta(3)-adrenoceptor antagonists in heart failure therapy. Cardiovasc Drug Rev. 2002;20(1):19–26. 12070531 10.1111/j.1527-3466.2002.tb00079.x

[25] Moniotte S Kobzik L Feron O Trochu JN Gauthier C Balligand JL . Upregulation of beta(3)-adrenoceptors and altered contractile response to inotropic amines in human failing myocardium. Circulation. 2001;103(12):1649–1655. 11273992 10.1161/01.cir.103.12.1649

[26] Zile MR Tanaka R Lindroth JR Spinale F Carabello BA Mirsky I . Left ventricular volume determined echocardiographically by assuming a constant left ventricular epicardial long-axis/short-axis dimension ratio throughout the cardiac cycle. J Am Coll Cardiol. 1992;20(4):986–993. 1388183 10.1016/0735-1097(92)90202-x

[27] Helak JW Reichek N . Quantitation of human left ventricular mass and volume by two-dimensional echocardiography: in vitro anatomic validation. Circulation. 1981;63(6):1398–1407. 7226486 10.1161/01.cir.63.6.1398

[28] Domenech RJ Hoffman JI Noble MI Saunders KB Henson JR Subijanto S . Total and regional coronary blood flow measured by radioactive microspheres in conscious and anesthetized dogs. Cir Res. 1969;25(5):581–596. 10.1161/01.res.25.5.581 5351326

[29] Suga H . Total mechanical energy of a ventricle model and cardiac oxygen consumption. Am J Physiol. 1979;236(3):H498–H505. 426086 10.1152/ajpheart.1979.236.3.H498

[30] Futaki S Nozawa T Yasumura Y Tanaka N Suga H . A new cardiotonic agent, OPC-8212, elevates the myocardial oxygen consumption versus pressure-volume area (PVA) relation in a similar manner to catecholamines and calcium in canine hearts. Heart Vessels. 1988;4(3):153–161. 3248983 10.1007/BF02058428

[31] How OJ Aasum E Larsen TS . Work-independent assessment of efficiency in ex vivo working rodent hearts within the PVA-MVO2 framework. Acta Physiol (Oxford, England). 2007;190(2):171–175. 10.1111/j.1748-1716.2007.01681.x 17394570

[32] Opie LH. Heart Physiology: From Cell to Circulation. Lippincott Williams & Wilkins; 2004.

[33] Demaison L Grynberg A . Cellular and mitochondrial energy metabolism in the stunned myocardium. Basic Res Cardiol. 1994;89(4):293–307. 7826305 10.1007/BF00795199

[34] Myrmel T Forsdahl K Larsen TS . Triacylglycerol metabolism in hypoxic, glucose-deprived rat cardiomyocytes. J Mol Cell Cardiol. 1992;24(8):855–868. 1433315 10.1016/0022-2828(92)91099-q

[35] Borst P Loos JA Christ EJ Slater EC . Uncoupling activity of long-chain fatty acids. Biochim Biophys Acta. 1962;62:509–518. 13871487 10.1016/0006-3002(62)90232-9

[36] Boardman NT Larsen TS Severson DL Essop MF Aasum E . Chronic and acute exposure of mouse hearts to fatty acids increases oxygen cost of excitation-contraction coupling. Am J Physiol Heart Circ Physiol. 2011;300(5):H1631–H1636. 21335471 10.1152/ajpheart.01190.2010

[37] Aasum E Hafstad AD Larsen TS . Changes in substrate metabolism in isolated mouse hearts following ischemia-reperfusion. Mol Cell Biochem. 2003;249(1-2):97–103. 12956404

[38] Randle PJ Garland PB Hales CN Newsholme EA . The glucose fatty-acid cycle. Its role in insulin sensitivity and the metabolic disturbances of diabetes mellitus. Lancet (London, England). 1963;1(7285):785–789. 10.1016/s0140-6736(63)91500-9 13990765

[39] Gauthier C Leblais V Kobzik L , et al. The negative inotropic effect of beta3-adrenoceptor stimulation is mediated by activation of a nitric oxide synthase pathway in human ventricle. J Clin Investig. 1998;102(7):1377–1384. 9769330 10.1172/JCI2191 PMC508985

[40] Dal Monte M Fornaciari I Nicchia GP Svelto M Casini G Bagnoli P . β3-adrenergic receptor activity modulates melanoma cell proliferation and survival through nitric oxide signaling. Naunyn Schmiedebergs Arch Pharmacol. 2014;387(6):533–543. 24599317 10.1007/s00210-014-0969-1

[41] Næsheim T How OJ Myrmel T . Hemodynamic effects of a soluble guanylate cyclase stimulator, riociguat, and an activator, cinaciguat, during NO-modulation in healthy pigs. J Cardiovasc Pharmacol Ther. 2021;26(1):75–87. 32662299 10.1177/1074248420940897 PMC7838342

[42] Chaudhry A MacKenzie RG Georgic LM Granneman JG . Differential interaction of beta 1- and beta 3-adrenergic receptors with Gi in rat adipocytes. Cell Signal. 1994;6(4):457–465. 7946969 10.1016/0898-6568(94)90093-0

[43] Thomas RF Holt BD Schwinn DA Liggett SB . Long-term agonist exposure induces upregulation of beta 3-adrenergic receptor expression via multiple cAMP response elements. Proc Natl Acad Sci USA. 1992;89(10):4490–4494. 1374904 10.1073/pnas.89.10.4490 PMC49108

[44] Bristow MR Ginsburg R Minobe W , et al. Decreased catecholamine sensitivity and beta-adrenergic-receptor density in failing human hearts. N Engl J Med. 1982;307(4):205–211. 6283349 10.1056/NEJM198207223070401

[45] Annane D Vignon P Renault A , et al. Norepinephrine plus dobutamine versus epinephrine alone for management of septic shock: a randomised trial. Lancet (London, England). 2007;370(9588):676–684. 10.1016/S0140-6736(07)61344-0 17720019

[46] Bistola V Arfaras-Melainis A Polyzogopoulou E Ikonomidis I Parissis J . Inotropes in acute heart failure: from guidelines to practical use: therapeutic options and clinical practice. Card Fail Rev. 2019;5(3):133–139. 31768269 10.15420/cfr.2019.11.2 PMC6848944

[47] Mebazaa A Nieminen MS Packer M , et al. Levosimendan vs dobutamine for patients with acute decompensated heart failure: the SURVIVE Randomized Trial. JAMA. 2007;297(17):1883–1891. 17473298 10.1001/jama.297.17.1883

[48] Tacon CL McCaffrey J Delaney A . Dobutamine for patients with severe heart failure: a systematic review and meta-analysis of randomised controlled trials. Intensive Care Med. 2012;38(3):359–367. 22160239 10.1007/s00134-011-2435-6

[49] Kjekshus JK Mjos OD . Effect of free fatty acids on myocardial function and metabolism in the ischemic dog heart. J Clin Investig. 1972;51(7):1767–1776. 5032525 10.1172/JCI106978 PMC292324

[50] Wallhaus TR Taylor M DeGrado TR , et al. Myocardial free fatty acid and glucose use after carvedilol treatment in patients with congestive heart failure. Circulation. 2001;103(20):2441–2446. 11369683 10.1161/01.cir.103.20.2441

[51] Gradinac S Coleman GM Taegtmeyer H Sweeney MS Frazier OH . Improved cardiac function with glucose-insulin-potassium after aortocoronary bypass grafting. Ann Thorac Surg. 1989;48(4):484–489. 2679462 10.1016/s0003-4975(10)66844-0

[52] Svedjeholm R Hallhagen S Ekroth R Joachimsson PO Ronquist G . Dopamine and high-dose insulin infusion (glucose-insulin-potassium) after a cardiac operation: effects on myocardial metabolism. Ann Thorac Surg. 1991;51(2):262–270. 1989542 10.1016/0003-4975(91)90798-u

